# Cuproptosis inhibits tumor progression and enhances cisplatin toxicity in ovarian cancer

**DOI:** 10.1096/fj.202500047R

**Published:** 2025-03-22

**Authors:** Qiaojian Zou, Yili Chen, Duo Liu, Qiqiao Du, Chunyu Zhang, Qiuwen Mai, Xiaojun Wang, Xiaoying Lin, Qianrun Chen, Mengxun Wei, Chudan Chi, Shuzhong Yao, Junxiu Liu

**Affiliations:** ^1^ Department of Obstetrics and Gynecology The First Affiliated Hospital of Sun Yat‐sen University Guangzhou China; ^2^ Department of Obstetrics and Gynecology Guangdong Provincial Clinical Research Center for Obstetrical and Gynecological Diseases Guangzhou China

**Keywords:** cisplatin, cuproptosis, Elesclomol, ovarian cancer, regulated cell death

## Abstract

Cuproptosis is a novel form of regulated cell death triggered by copper ion and copper ionophore. While cuproptosis has been actively explored as a potential target for cancer therapy, its role in ovarian cancer (OC) still remains unclear. In this study, we demonstrate that cuproptosis inhibits OC cell proliferation, migration, and invasion through FDX1 regulation and suppresses tumor growth in a mouse model. We also confirm that cuproptosis enhances OC sensitivity to cisplatin treatment both in vivo and in vitro. Moreover, our findings reveal that cuproptosis affects cholesterol biosynthesis in OC cells, with cholesterol playing a crucial role in its cytotoxic effect. Taken together, our results elucidate the effect of cuproptosis in OC and suggest it as a promising therapeutic strategy.

## Graphical abstract

Cuproptosis, a novel form of regulated cell death, inhibits ovarian cancer (OC) proliferation, migration, and invasion through FDX1 regulation. The combination treatment of cuproptosis also enhances OC sensitivity to cisplatin. Moreover, our results reveal that cuproptosis affects cholesterol biosynthesis in OC cells, with cholesterol playing a crucial role in its cytotoxic effect. This study elucidates the effect of cuproptosis in OC and suggests it as a promising therapeutic strategy.

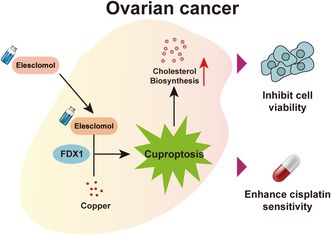

## 1 INTRODUCTION

Ovarian cancer (OC) is the eighth most common female malignancy worldwide. In 2022, over 320 000 women were newly diagnosed, and more than 200 000 patients died from OC.^1^ Ovaries are located deep in the female pelvic cavity; thus, early‐stage OC often shows no specific symptoms, leading to 75% of diagnoses occurring at an advanced stage (FIGO stage III/IV), which results in a poor prognosis.^2^ Therefore, effective strategies for early diagnosis and precise treatment of OC become extremely important.

Recently, a novel form of regulated cell death called cuproptosis was discovered by Tsvetkov et al.^3^ Unlike other types of cell death such as apoptosis, ferroptosis, necroptosis, and pyroptosis, cuproptosis is mainly induced by copper ion in the presence of copper ionophore. Under the influence of copper ionophores such as Elesclomol, the cuproptosis key regulatory gene ferredoxin 1 (FDX1) reduces Cu^2+^ to its more toxic form Cu^1+^ and directly binds to lipoylated proteins of the pyruvate dehydrogenase complex. With the assistance of LIAS, Cu^1+^ induces the oligomerization of lipoylated DLAT, which increases HSP70 abundance, disrupts the tricarboxylic acid (TCA) cycle, and impairs mitochondrial respiration, and ultimately leads to cell death.

Increasing research has focused on the mechanism of cuproptosis in malignant tumors. Li et al. constructed a cuproptosis‐related lncRNA signature in hepatocellular carcinoma and validated its effectiveness in both cells and clinical tissues.^4^ Yang et al. demonstrated that 4‐octyl itaconate can inhibit aerobic glycolysis and promote cuproptosis in colorectal cancer.^5^ Guo et al. gained the first insight into combining nanomedicine that can induce cuproptosis with αPD‐L1 to enhance cancer therapy.^6^ Sun et al. discovered that lactylation of METTL16 could promote cuproptosis and improve the therapeutic efficacy of Elesclomol in gastric cancer.^7^ Accumulating evidence suggests that cuproptosis could be a promising target for cancer treatment. In OC, Li et al. identified a novel cuproptosis‐related lncRNAs signature that effectively predicts prognosis and sensitivity to antineoplastic drugs.^8^ Wang et al. constructed a cuproptosis‐related prognostic model for OC patients and elucidated the role of WASF2 in cuproptosis resistance.^9^ Additionally, Li et al. utilized single‐cell sequencing to analyze cuproptosis‐associated genes in OC and provided insights into predicting responses to immunotherapy.^10^ However, most of these studies are based on bioinformatic analysis, and the precise mechanisms underlying cuproptosis in OC remain largely unclear.

This study is the first to evaluate the anticancer potential of cuproptosis in OC. In this study, we revealed that cuproptosis could significantly inhibit OC cell viability through the regulation of FDX1. Furthermore, we demonstrated that cuproptosis could enhance the killing effect of cisplatin on OC both in vivo and in vitro. This finding may provide an effective therapeutic alternative for OC patients.

## 2 MATERIALS AND METHODS

### 2.1 Reagents

All reagents and antibodies used in this study are listed in Table S1. Elesclomol was diluted by dimethyl sulfoxide (DMSO), and cisplatin was diluted with saline or PBS under ultrasonic vibration. All other reagents were diluted with double‐distilled water.

### 2.2 Clinical specimens

We collected 30 normal ovary tissues and 29 OC tissues between May 2023 and November 2023 from the First Affiliated Hospital of Sun Yat‐sen University (Guangzhou, China). Normal ovary tissues were obtained from patients who underwent adnexectomy under nonmalignant conditions. None of the OC patients had received neoadjuvant therapy prior to surgery. All specimens were immediately frozen in liquid nitrogen during surgery and stored at −80°C until RNA isolation. This study was approved by the Ethical Review Committee of the First Affiliated Hospital of Sun Yat‐sen University for the use of these clinical tissues for research purposes. All samples were obtained in accordance with the Declaration of Helsinki, and each patient signed written informed consent for all procedures.

### 2.3 Cell culture and transfection

The human OC cell lines SKOV‐3 and A2780 were purchased from the American Type Culture Collection (ATCC) and cultured in complete McCoy's 5A and DMEM medium, supplemented with 10% fetal bovine serum and 1% penicillin/streptomycin. Both cell lines were cultured in a humidified atmosphere with 5% CO_2_ at 37°C.

To knock down or overexpress FDX1, we purchased siRNA from Genepharma (Shanghai, China) and plasmid from OBiO (Shanghai, China). When the cell density reached approximately 60%, siRNA and plasmid transfections were carried out using Lipofectamine RNAiMAX and Lipofectamine 3000 (Invitrogen, USA), following the manufacturer's instructions. Further experiments were conducted 48 h after transfection. The siRNA sequences used are listed in Table S2.

### 2.4 Cell proliferation, migration, invasion, and apoptosis assays

SKOV‐3 or A2780 cells were seeded into 96‐well plates at a density of 2000 or 5000 cells per well. CCK‐8 solution was carefully added to the plates (10 μL/well). Optical density at 450 nm was measured by a fluorescence microplate reader after incubating at 37°C. For the colony formation assay, SKOV‐3 or A2780 cells were digested and replanted into six‐well plates at a density of 1500 or 5000 cells per well. After 7 days of culture, cells were fixed with 4% paraformaldehyde and stained with 0.1% crystal violet. Each well was photographed separately.

We used a 24‐well plate Transwell system to assess cell invasion ability. After precoating the upper chamber with 50 μL of Matrigel, 2.5 × 10^4^ of SKOV‐3 and 1.5 × 10^5^ of A2780 cells in 200 μL of serum‐free medium were then seeded into the upper chamber of 8 μm Transwell inserts and incubated in a 10% FBS complete culture medium for 24 h. Next, cells were fixed with 4% paraformaldehyde, stained with 0.1% crystal violet, and photographed and counted under a microscope. For the wound healing assay, culture inserts (ibidi, German) were used to create scratches among cells. A total of 2.5 × 10^4^ SKOV‐3 cells were seeded into each quadrant of the inserts in complete culture medium. Inserts were removed after 12 h, and cells were then cultured in serum‐free medium. Photographing was processed under a microscope for comparing wound healing abilities.

Apoptosis assays were performed as previously described.^11^ Annexin V‐FITC/PI Apoptosis Kit (Elabscience, China) was used according to the manufacturer's instructions. SKOV‐3 and A2780 cells were planted into six‐well plates and cultured with Elesclomol‐Cu for 24 h. The cell apoptotic rate was measured 48 h later by a flow cytometer (Beckman CytoFLEX) and analyzed by CytExpert software. Each assay was repeated three times.

### 2.5 Xenograft animal model

Female BALB/c nude mice (4–6 weeks of age) were purchased from Vital River Laboratory (Guangdong, China) and raised under SPF conditions. 2 × 10^6^ of SKOV‐3 cells were subcutaneously injected into the back of nude mice with 150 μL of PBS to create the subcutaneous xenograft tumor model. After 7 days, nude mice were randomly divided into different groups and injected intraperitoneally with drugs twice a week. The Elesclomol treatment lasted 35 days with 10 injections, while the combination of Elesclomol and cisplatin lasted 21 days with five injections due to the side effects of cisplatin. All mice were anesthetized and sacrificed after repetitive injections. Tumor volume was measured by caliper and calculated using the following formula: volume (mm^3^) = (length [mm]) × (width [mm])^2^ × 0.52.^12^ All experimental procedures were approved by the Institutional Animal Ethics Committee of Zhiyuan, Guangdong, China (Approval Number: IAEC‐2023050101).

### 2.6 Immunohistochemistry (IHC) and hematoxylin–eosin (HE) staining

IHC and HE staining were performed as previously described.^11^ The primary antibodies and their dilution ratios used in this study are listed in Table S1. Antigen retrieval was performed by heating in Tris‐EDTA buffer (pH 9.0) at 120°C for 2 min. The IHC scores of FDX1 (range from 0 to 3) were evaluated by H score.^13^ The total number of cells in each field and the number of cells stained at each intensity were counted. The H score was calculated as follows: [(% of week staining × 1) + (% of moderate staining × 2) + (% of strong staining × 3)]/100. For Ki‐67, the percentage of positively stained cells was evaluated. All tissue sections were observed under an optical scanner.

### 2.7 RNA isolation, quantitative real‐time PCR (qRT‐PCR), reverse transcription‐PCR (RT‐PCR), and western blotting

Total RNAs were extracted from cells and tissues using the SteadyPure Universal RNA Extraction Kit (Accurate Biology, China) according to the manufacturer's instructions. RT‐PCR and qRT‐PCR were performed using the Evo M‐MLV RT Mix Kit and SYBR Green Premix Pro Taq HS qPCR Kit (Accurate Biology, China) as described previously.^14^ Data obtained from qRT‐PCR were analyzed based on the 2^−ΔΔCT^ method. All primers were synthesized by Generay Biotech (Shanghai, China) and primer sequences are given in Table S3. Western blotting was performed as previously described.^15^ The dilution ratios of primary antibodies were given in Table S1.

### 2.8 RNA sequencing

Total RNA was extracted by Trizol reagent (Invitrogen) and stored at −80°C. Oligo(dT) magnetic beads were used to isolate mRNA from total RNA. Construction of cDNA libraries before sequencing was conducted with the assistance of OBiO Scientific Services (Shanghai, China), following the manufacturer's instructions. The quality of the libraries was assessed using Agilent 2200, and sequencing was performed on the Illumina NovaSeq 6000 platform on a 150‐bp paired‐end run.

### 2.9 Copper detection

Eyeballs of nude mice were removed under anesthesia to collect blood. Serum was separated for copper detection using the copper detection kit (Leagene Biotechnology, Beijing, China). We mixed serum, copper‐acidizing buffer, and protein removal solution and then centrifuged for 10 min at 3000 *g*. Then, we added a mixture containing 140 μL of Cu assay buffer, 10 μL of color solution, and 100 μL of samples in a 96‐well plate. The samples were incubated at room temperature for 20 min and measured the absorbance at 620 nm.

### 2.10 Cholesterol detection

Cholesterol level was measured by the total cholesterol content assay kit (Boxbio, Beijing, China). OC cells were counted and dissolved in isopropanol before ultrasonic lysis. The extract was then centrifuged for 10 min at 10 000 *g*. Meanwhile, we prepared the standard curve with the cholesterol standard. We added a mixture containing 180 μL of cholesterol assay buffer and 20 μL of samples in a 96‐well plate. The samples were incubated at 37°C for 15 min and measured the absorbance at 500 nm.

### 2.11 Statistical analysis

Statistical analysis was performed with SPSS 23.0 statistical software and GraphPad Prism version 10.1.2. The independent sample *t*‐test was used to compare differences between two groups. One‐way analysis of variance was applied to assess differences among multiple groups. The half‐maximal inhibitory concentration (IC_50_) was calculated by nonlinear regression of GraphPad Prism. In all cases, *p* < .05 was considered statistically significant.

## 3 RESULTS

### 3.1 Copper is essential for OC cell cuproptosis

**FIGURE 1:**
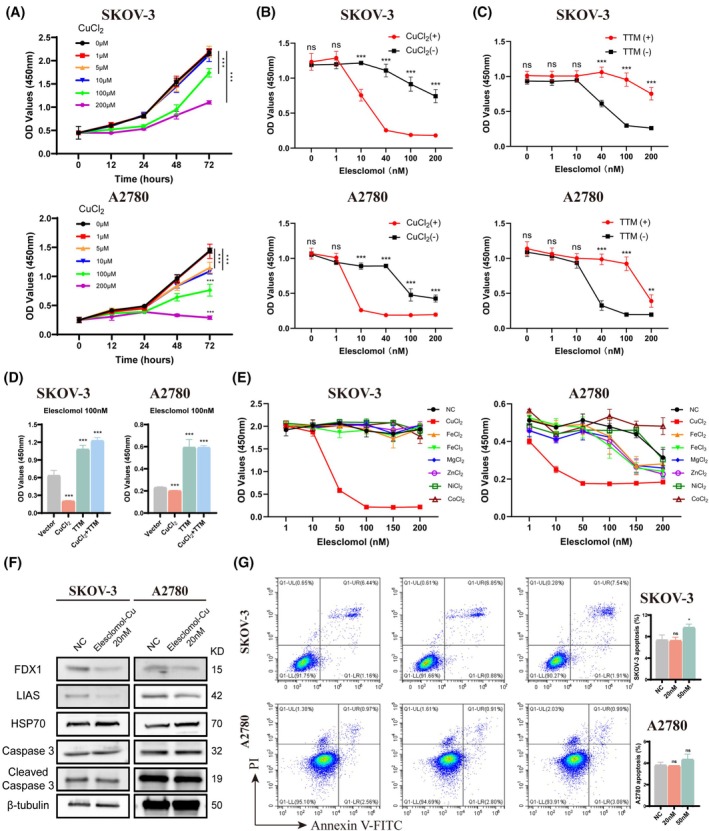
Copper is essential for OC cell cuproptosis. (A) The proliferative abilities of SKOV‐3 and A2780 cells were measured by CCK‐8 assay after exposure to different concentrations of copper. (B–D) OC cells were treated with different concentrations of Elesclomol alone or combined with 1 μM CuCl_2_ or 1 μM TTM for 48 h and measured the viability by CCK‐8 assay. (E) Viability of OC cells after treatment with Elesclomol with 10 μM of indicated metals. (F) Expression of FDX1, LIAS, HSP70, Caspase 3, and Cleaved Caspase 3 in OC cells treated with 20 nM of Elesclomol‐Cu for 24 h. (G) Flow cytometry analysis for Annexin V‐FITC and PI staining in OC cells treated with 20 or 50 nM of Elesclomol‐Cu for 24 h. TTM, tetrathiomolybdate. ns, no significance. **p < .01; ***p < .001.

A certain range of copper in plasma plays an important role in body function.^16^ To examine whether copper and cuproptosis impact the development of OC, we added various concentrations of cupric chloride (CuCl_2_) to SKOV‐3 and A2780 cell lines. We observed that 1 μM of exogenous copper had no apparent effect on the proliferation of both cells. However, copper concentrations of 5 μM in A2780 and 100 μM in SKOV‐3 could significantly inhibit cell growth (Figure 1A). Besides, even after 72 h of continuous stimulation with 200 μM copper, only a portion of SKOV‐3 cells exhibited morphological change and death (Figure S1A). These results indicated that small amounts of copper do not affect OC cell growth.

Previous studies have declared that copper could induce cuproptosis when accompanied with Elesclomol, which acts as a copper ionophore.^3^ Our results showed that 1 μM of exogenous copper had no apparent effect on OC cells. However, when combined with Elesclomol, it severely impaired cell viability in a dose‐dependent manner, with a greater effect than Elesclomol alone (Figure 1B). Meanwhile, this combined effect could be partially reversed by chelation of copper with tetrathiomolybdate (TTM) (Figure 1C). Our results revealed that copper could efficiently inhibit OC cell growth in combination with Elesclomol (Figures 1D and S1B). We also tested other trace elements including iron, magnesium, zinc, nickel, and cobalt and discovered that only copper together with Elesclomol caused obvious damage to OC cells (Figure 1E). Next, western blot analysis also revealed that the Fe–S cluster proteins FDX1 and LIAS were decreased after Elesclomol‐Cu treatment, while the expression of HSP70 was increased, which was consistent with the characteristics of cuproptosis (Figure 1F). Besides our results indicated that although Elesclomol‐Cu induced cell death, it did not elevate Cleaved Caspase 3 levels in OC cells. Similarly, flow cytometry results further showed that, except for a slight increase in apoptosis levels in SKOV‐3 cells treated with 50 nM Elesclomol‐Cu, those cell death was primarily not associated with apoptosis (Figure 1G). Collectively, these findings confirmed that copper is crucial for inducing cuproptosis in OC cells with the cooperation of Elesclomol.

### 3.2 Pulse treatment of Elesclomol‐Cu damages OC cell viability

**FIGURE 2:**
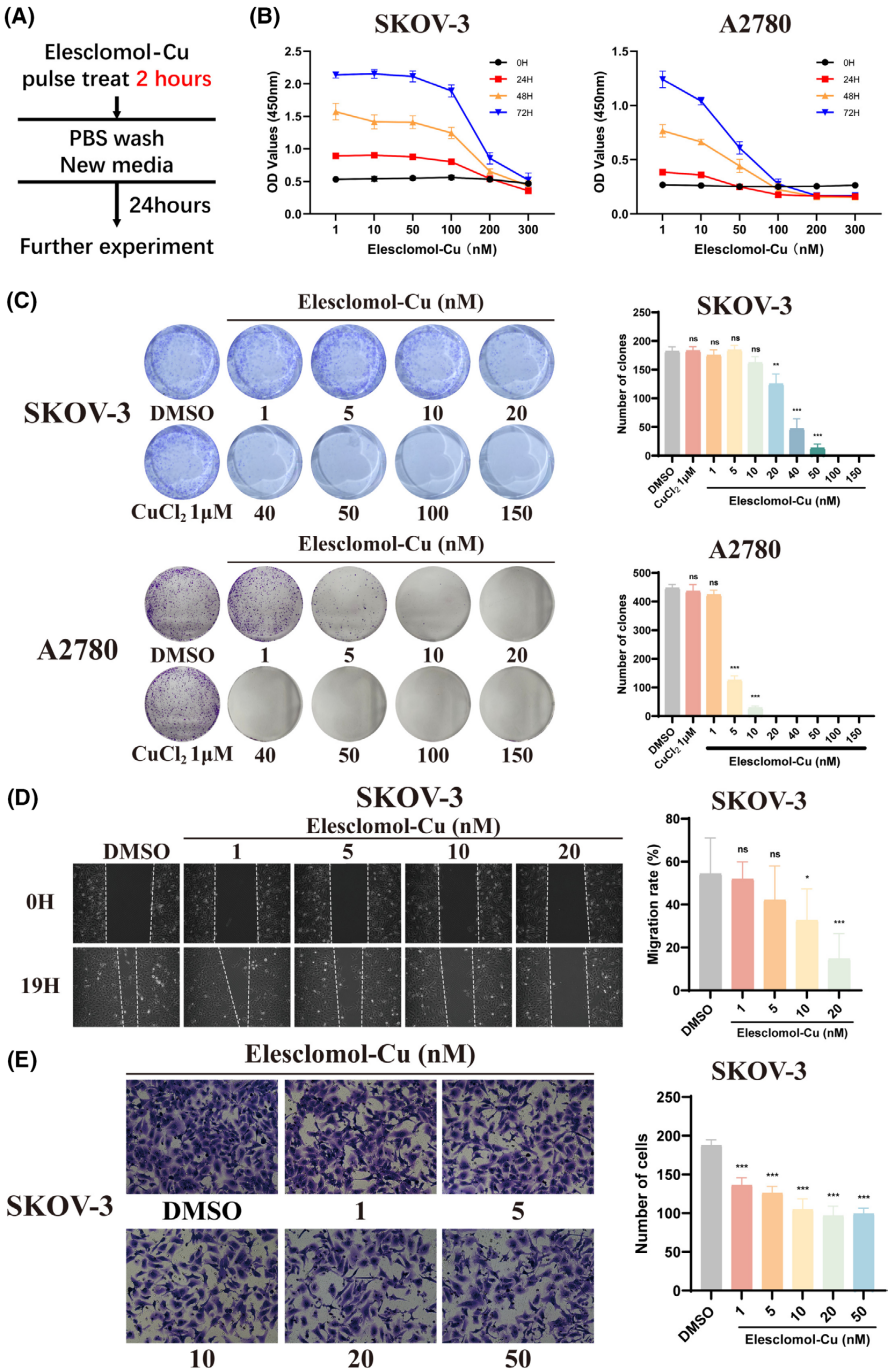
Pulse treatment of Elesclomol‐Cu damages OC cell viability. (A) OC cells were pulse treated with Elesclomol‐Cu for 2 h before further experiments. (B, C) CCK‐8 and colony formation assays evaluated the proliferative abilities for OC cells with pulse treatment of Elesclomol‐Cu. (D) Migration abilities were investigated by wound healing assay of OC cells. Original magnification, × 100. (E) Transwell assays were performed to investigate the effects of Elesclomol‐Cu pulse treatment on the invasion abilities of OC cells. Original magnification, × 200. ns, no significance. *p < .05; **p < .01; ***p < .001.

To further investigate the effect of cuproptosis on OC cells, we treated them shortly with Elesclomol‐Cu for 2 h (Figure 2A). The CCK‐8 assay showed that pulse treatment inhibited the proliferative ability of both OC cells, especially A2780 (Figure 2B). The colony formation assay indicated that 1 μM copper did not affect cell viability, but when combined with Elesclomol, it significantly reduced the ability of cells to form colonies (Figure 2C). Similarly, wound healing and Transwell assays also demonstrated that pulse treatment with Elesclomol‐Cu impaired the migration and invasion abilities of OC cells in a dose‐dependent manner (Figure 2D,E). Taken together, these results indicated that even a short‐term exposure to Elesclomol‐Cu could induce cuproptosis and significantly damage the viability of OC cells, including their proliferation, migration, and invasion abilities.

### 3.3 FDX1 enhances OC cell cuproptosis

**FIGURE 3:**
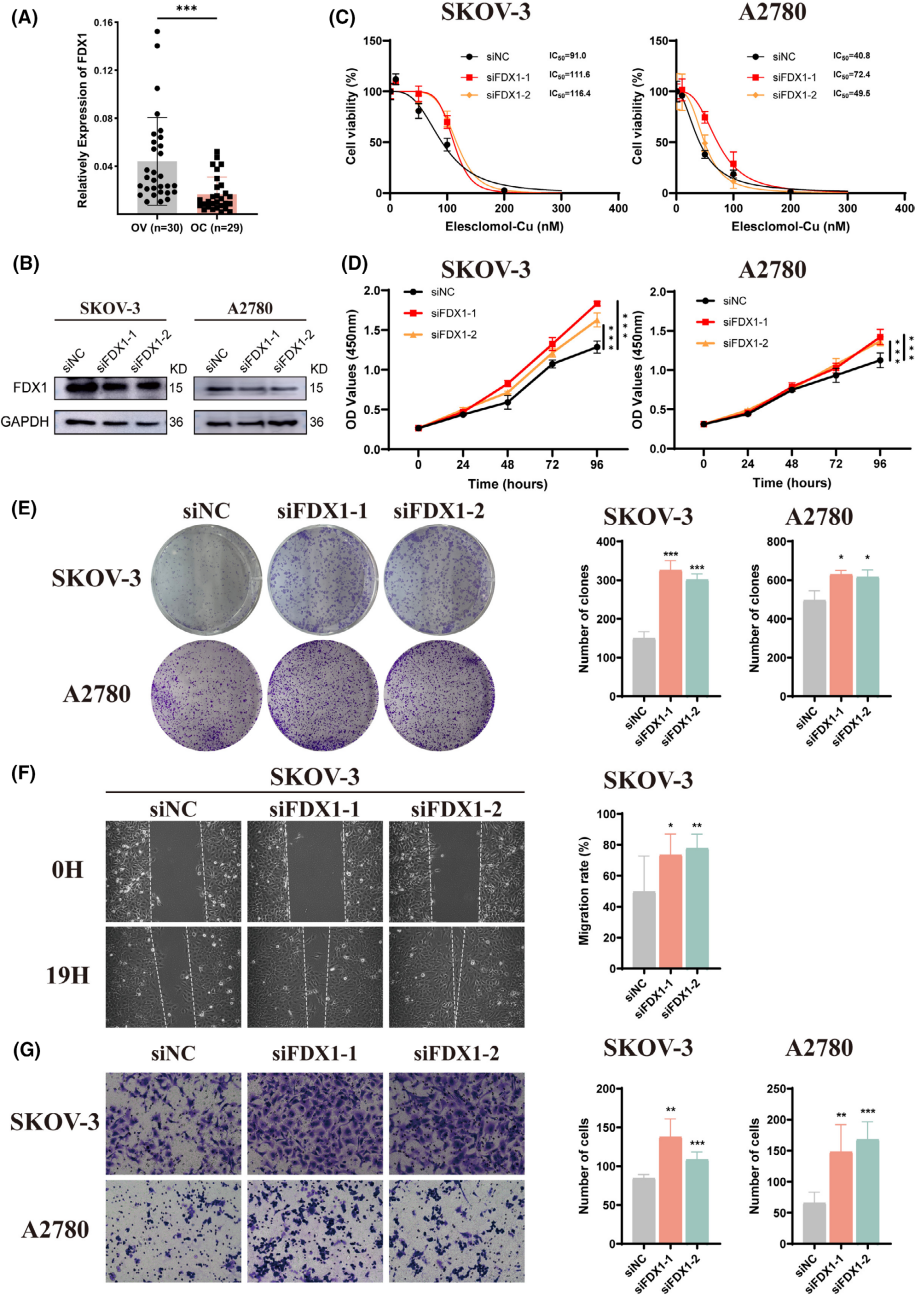
Knockdown of FDX1 inhibits OC cell cuproptosis. (A) qRT‐PCR analysis of FDX1 expression in normal ovary tissues and OC samples. (B) Expression of FDX1 in SKOV‐3 and A2780 cells treated with siRNA. (C) FDX1‐knockdown OC cells were exposed to different concentrations of Elesclomol‐Cu for 24 h and measured IC_50_ by CCK‐8 assay. (D, E) Proliferative capacity of FDX1 knockdown OC cells was determined by CCK‐8 and colony formation assay after co‐cultured with 5 nM Elesclomol‐Cu. (F, G) Wound healing and Transwell assays were performed to investigate the migration and invasion abilities of OC cells after knockdown of FDX1 and exposure to 5 nM Elesclomol‐Cu. Results were the mean from three independent experiments. Data were represented as mean ± SEM. OV, normal ovary tissue. ns, no significance. *p < .05; **p < .01; ***p < .001.

To better understand the relationship between OC and the key cuproptosis regulator FDX1, we first tested the expression of FDX1 in normal ovary and OC tissues collected from our hospital. Our results showed that the mRNA level of FDX1 was significantly lower in OC tissues (Figure 3A). Next, we utilized siRNA to knock down FDX1 in OC cells (Figure 3B). When treated with different concentrations of Elesclomol‐Cu, we observed that FDX1 interference significantly rescued copper‐dependent cell death (Figure 3C). Additionally, we conducted a series of functional assays to evaluate FDX1 and cuproptosis in OC cells. After FDX1 knockdown, we subsequently treated OC cells with 5 nM Elesclomol‐Cu for 2 h. We noticed that FDX1‐knockdown OC cells were more resistant to the reduction in proliferation and colony formation abilities (Figure 3D,E). Meanwhile, knockdown of FDX1 also prevented the impairment of cell migration and invasion abilities caused by cuproptosis (Figure 3F,G).

**FIGURE 4:**
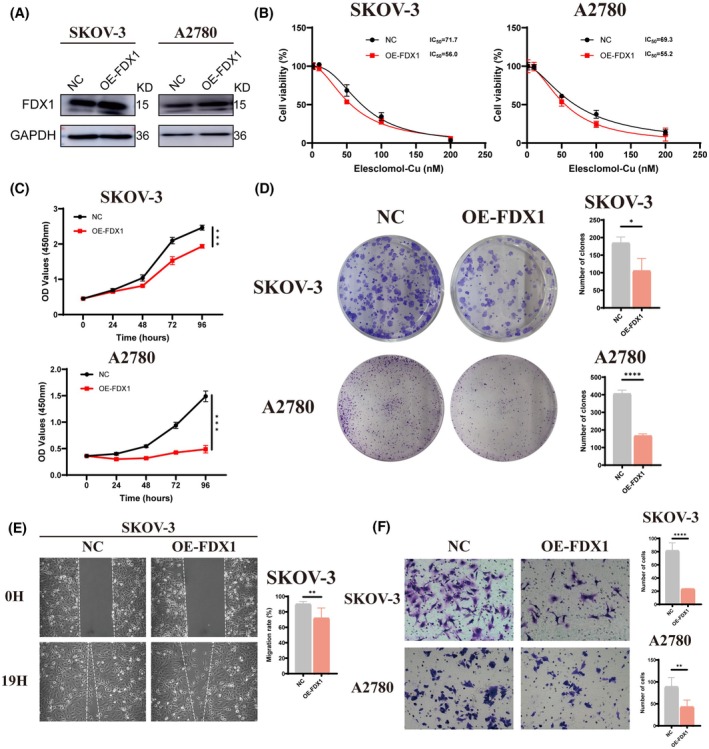
FDX1 enhances OC cell cuproptosis. (A) Expression of FDX1 in OC SKOV‐3 and A2780 cells treated with plasmid. (B) FDX1‐overexpressed OC cells were exposed to Elesclomol‐Cu for 24 h and measured IC_50_ by CCK‐8 assay. (C, D) Proliferative capacity of FDX1 overexpression OC cells was determined by CCK‐8 and colony formation assays after co‐culturing with 5 nM Elesclomol‐Cu. (E, F) Wound healing and Transwell assays were performed to investigate the migration and invasion abilities of OC cells after overexpression of FDX1 and exposure to 5 nM Elesclomol‐Cu. Results were the mean from three independent experiments. Data were represented as mean ± SEM. ns, no significance. *p < .05; **p < .01; ***p < .001; ****p < .0001.

Again, we also overexpressed FDX1 in OC cells (Figure 4A). As expected, FDX1 overexpression increased the sensitivity of OC cells to Elesclomol‐Cu treatment (Figure 4B). Following pulse treatment with 5 nM Elesclomol‐Cu, we observed that FDX1‐overexpressing OC cells showed significant impairment in proliferation and colony formation abilities (Figure 4C,D). In addition, the overexpression of FDX1 also enhanced the inhibitory effects of cuproptosis on migration and invasion abilities (Figure 4E,F). Collectively, our findings demonstrated that FDX1 could enhance OC cell sensitivity to cuproptosis and cause impairment to their proliferation, migration, and invasion abilities.

### 3.4 Cuproptosis inhibits OC growth in vivo

**FIGURE 5:**
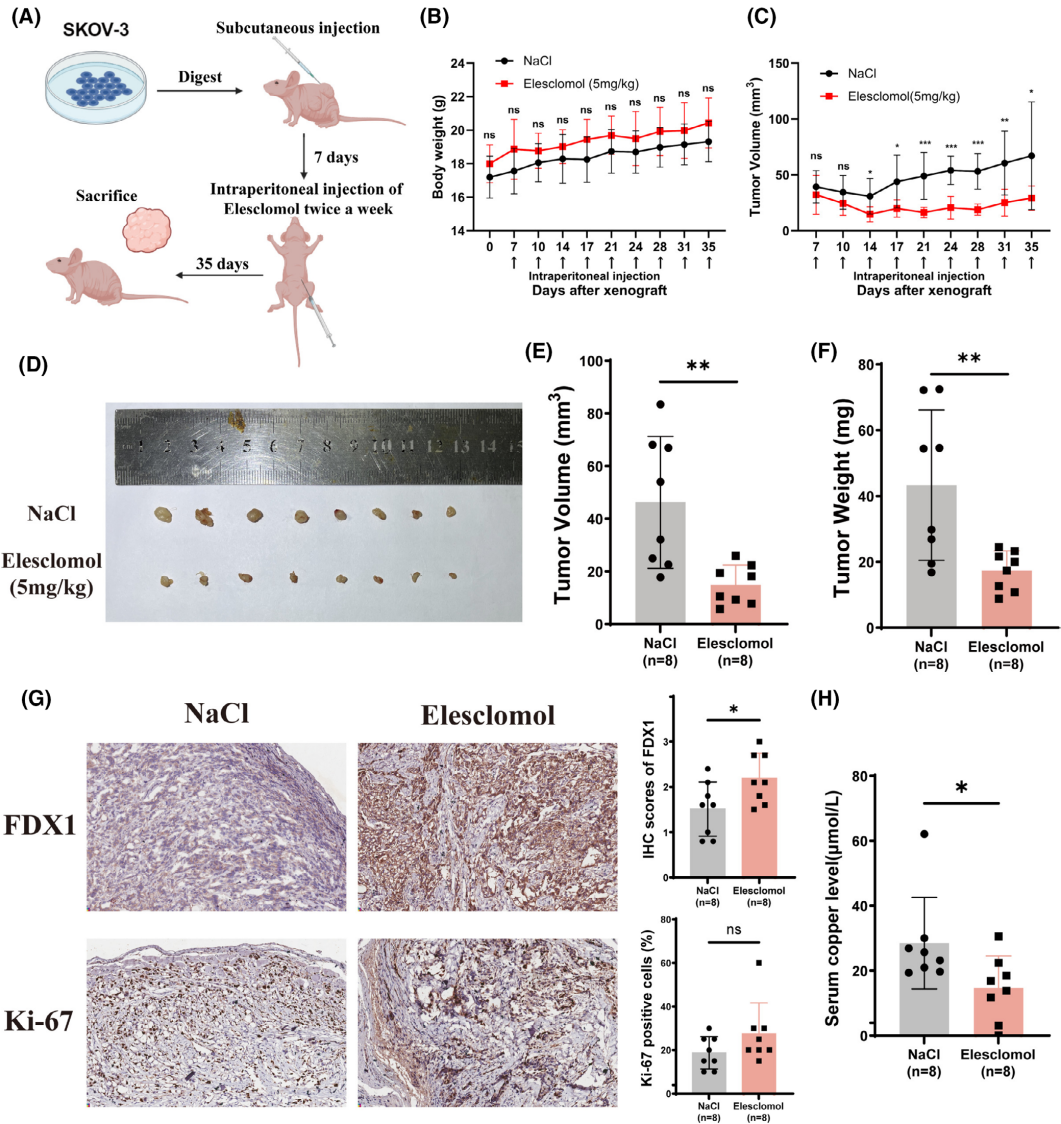
Cuproptosis inhibits OC growth in vivo. (A) Schematic illustration of subcutaneous xenograft tumor model. (B, C) Monitoring the body weight and tumor volume of nude mice during the experiment. (D) Representative image of subcutaneous tumors of indicated groups (*n* = 8). (E, F) Scatter plots showed the tumor volume and weight in different groups. (G) The expression of FDX1 and Ki‐67 protein in tumors was detected by IHC. (H) The serum copper level of different groups of nude mice. ns, no significance. *p < .05; **p < .01; ***p < .001.

Next, we further investigated the potential effect of cuproptosis on OC by animal experiments. A subcutaneous xenograft tumor model was established in the female BALB/c nude mice with SKOV‐3 cells (Figure 5A). After 10 continuous intraperitoneal injections of Elesclomol or saline control twice a week, we found that regular Elesclomol treatment significantly reduced tumor volume without affecting the mice's body weight (Figure 5B,C). Directly measuring the volume and weight of tumors also got similar results (Figure 5D–F). Besides we evaluated the subcutaneous tumors by IHC staining and discovered that FDX1 was highly expressed in Elesclomol group, but the expression of the proliferation marker Ki‐67 showed no difference between the two groups (Figure 5G). Meanwhile, we also extracted the blood of mice and found serum copper levels were remarkably lower in the Elesclomol group (Figure 5H). Our results indicated that the inhibition ability of Elesclomol to OC tumor growth might be closely related to cuproptosis. Taken together, these data revealed that repetitive exposure to Elesclomol could suppress OC tumor growth in vivo.

### 3.5 Cuproptosis enhances the killing effect of cisplatin on OC

**FIGURE 6:**
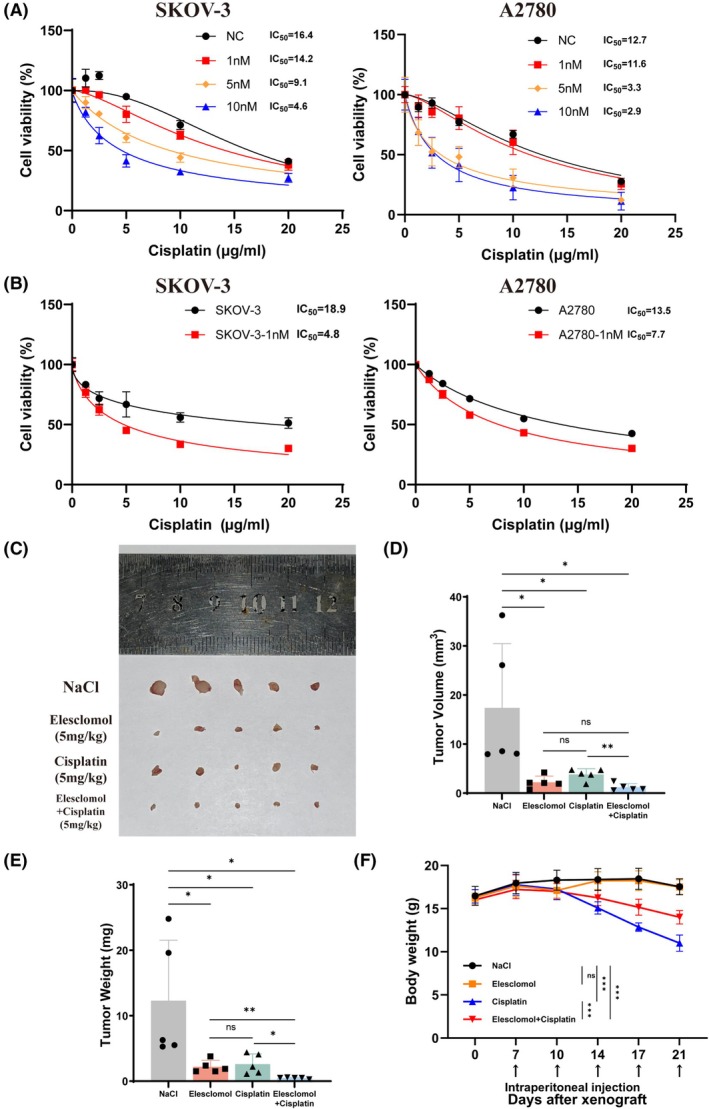
Cuproptosis enhances the killing effect of cisplatin on OC cells. (A) Cell viability and IC_50_ were assessed in OC SKOV‐3 and A2780 cells after receiving 24 h of low concentrations of Elesclomol‐Cu and 24 h of cisplatin sequential therapy. (B) OC cells were cultured in a low concentration of 1‐nM Elesclomol‐Cu for 7 days followed by treatment with cisplatin for 24 h. (C) Representative image of subcutaneous tumors of indicated groups (*n* = 5). (D, E) Scatter plots showed the tumor volume and weight in different groups. (F) Monitoring the body weight of nude mice during the experiment. ns, no significance. *p < .05; **p < .01; ***p < .001.

Our results showed that cuproptosis in OC cells primarily occurred in the presence of copper. Also, Tsvetkov et al. demonstrated that cuproptosis depended largely on intracellular copper accumulation, and altering the expression of key copper transport proteins such as SLC31A1, ATP7A, and ATP7B could remarkably affect cellular sensitivity to cuproptosis.^3^ Additionally, previous studies have showed that copper transport protein simultaneously influences the influx and efflux of not only copper but also platinum, and there is a competitive inhibition effect between their transport.^17^ Therefore, we hypothesized that cuproptosis might influence the transport balance of copper and platinum, thereby altering the sensitivity of OC cells to platinum‐based drugs. To test this, we treated OC cells with Elesclomol‐Cu for 24 h and observed a decrease in the IC_50_ of subsequent cisplatin treatment (Figure 6A). Meanwhile, we cultured OC cells with a low concentration of 1 nM Elesclomol‐Cu for 1 week to assess the effects of long‐term exposure. As expected, the IC_50_ of cisplatin also decreased under this treatment (Figure 6B). These results suggested that cuproptosis could enhance the sensitivity of OC cells to cisplatin in vitro.

We then established a subcutaneous xenograft tumor model in female BALB/c nude mice to evaluate the effect of cuproptosis and cisplatin. After 3 weeks of continuous intraperitoneal injections, both the cisplatin and Elesclomol treatment groups showed reduced tumor volumes compared to the control group, with the combination of two drugs resulting in a more significant decrease (Figure 6C,D). Similar tumor weight also showed a similar trend across four groups (Figure 6E). Then, we further evaluated the commonly reported side effects of cisplatin exposure, including weight loss and kidney injury.^18^ Continuous monitoring revealed that Elesclomol alone did not cause body weight loss in mice, while cisplatin treatment resulted in severe weight loss. Interestingly, although the combined treatment group also experienced weight loss, it was less severe than that of the cisplatin monotherapy group (Figure 6F), suggesting that Elesclomol treatment could partially reverse the weight loss caused by cisplatin. In addition, we assessed the kidney injury in each group by HE staining. Cisplatin caused significant damage, including renal cortex thinning, medulla telangiectasia, collecting tubule damage, renal papillary and hilus atrophy, and renal hilus vasodilation, but Elesclomol did not. Notably, the combination usage of Elesclomol mitigated most of the kidney injury caused by cisplatin, except for renal medulla telangiectasia (Figure S2). Taken together, these results suggested that cuproptosis could enhance the cytotoxic effect of cisplatin on OC in vitro and in vivo and partially alleviate cisplatin‐induced side effects of weight loss and kidney injury.

### 3.6 Cuproptosis influences cholesterol homeostasis of OC cells

**FIGURE 7:**
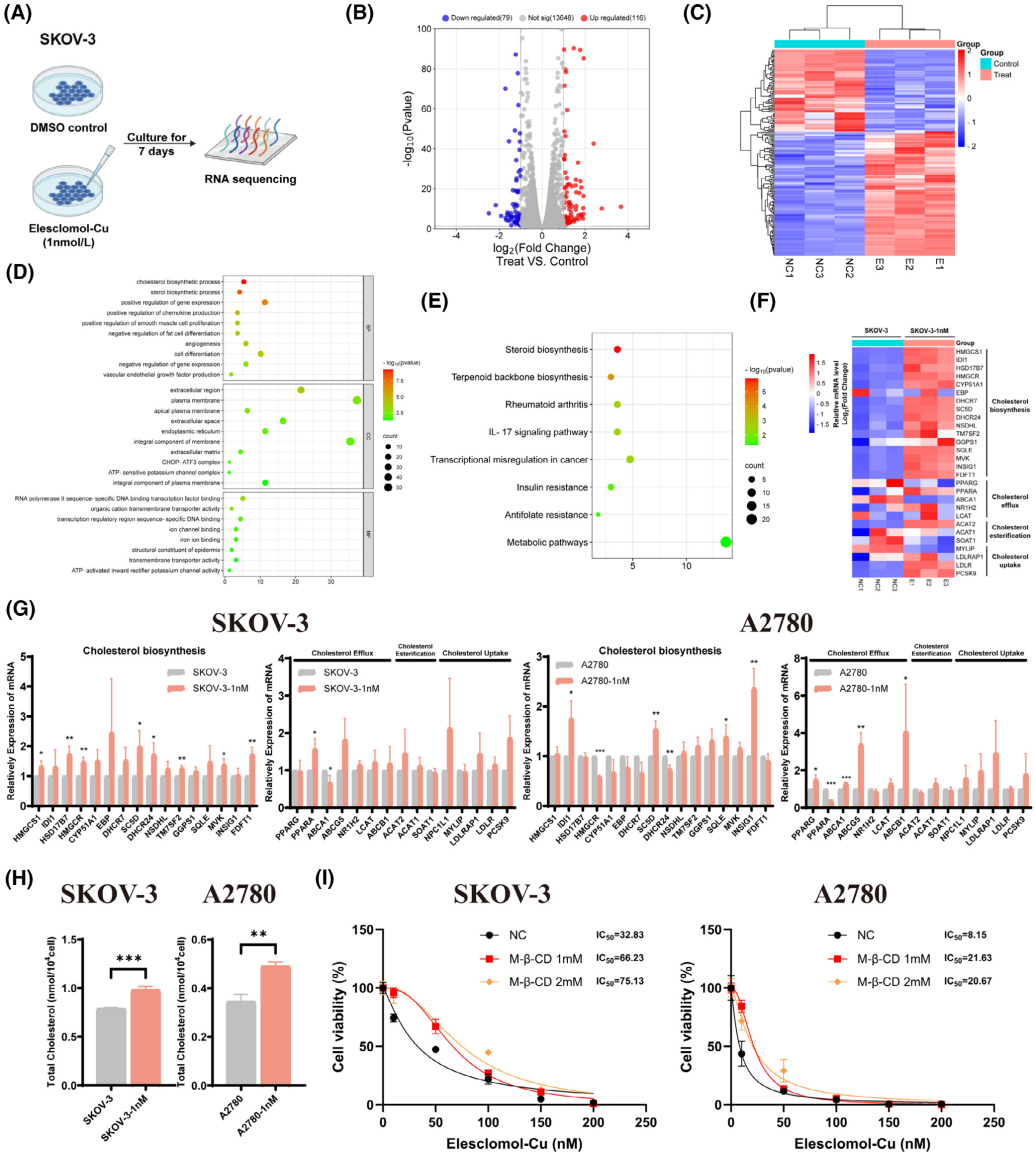
Cuproptosis influences cholesterol homeostasis of OC cells. (A) SKOV‐3 cells were cultured with 1 nM of Elesclomol‐Cu or DMSO for 7 days and subsequently performed RNA sequencing. (B, C) The differentially expressed genes between two groups were shown as the volcano plot and heat map matrix. The threshold was set as Log_2_|fold change| ≥1 and *p* < .05. (D, E) GO and KEGG analyses of all differentially expressed genes. (F) Heat map of important cholesterol homeostasis genes from RNA sequencing. (G) Expression of cholesterol homeostasis genes after treatment with 1 nM Elesclomol‐Cu or DMSO for 7 days. (H) OC cells were cultured with 1 nM Elesclomol‐Cu or DMSO for 7 days before assessing the total cholesterol level. (I) IC_50_ was assessed in OC cells after receiving 24 h of Elesclomol‐Cu and 30 min of M‐β‐CD sequential treatment. M‐β‐CD, Methyl‐β‐cyclodextrin. ns, no significance. *p < .05; **p < .01; ***p < .001.

To further investigate the potential targets of cuproptosis, we cultured SKOV‐3 with a low concentration of 1 nM Elesclomol‐Cu for 7 days and performed RNA sequencing (Figure 7A). We identified 195 differentially expressed genes (DEGs) by the threshold of Log_2_|fold change| ≥1 and *p* < .05, with 116 genes upregulated and 79 downregulated (Figure 7B,C). GO and KEGG pathway analysis of DEGs revealed significant enrichment in several metabolic‐related pathways, particularly those related to steroid and cholesterol biosynthesis (Figure 7D,E). Therefore, we further analyzed the expression of cholesterol homeostasis genes, including those involved in cholesterol biosynthesis, efflux, esterification, and uptake, and noticed that several cholesterol biosynthesis‐associated genes were significantly upregulated after Elesclomol‐Cu treatment (Figure 7F). The results of qRT‐PCR also confirmed an obvious increase in mRNA levels of these genes in OC cells (Figure 7G), indicating the activation of cholesterol biosynthesis.

To further verify these findings, we measured the cholesterol level in OC cells and found an increase in total cholesterol concentration in both SKOV‐3 and A2780 cells following cuproptosis induction (Figure 7H). Moreover, we treated OC cells with methyl‐β‐cyclodextrin (M‐β‐CD) to remove cholesterol. As shown in Figure 7I, cholesterol depletion reduced the lethal effect of cuproptosis on OC cells. Collectively, our findings showed that cuproptosis could affect cholesterol homeostasis in OC cells, with cholesterol playing a crucial role in its cytotoxic effect.

## 4 DISCUSSION

As the third most abundant trace element in the human body, copper plays a pivotal role in the normal operation of many physiological processes, including the biotransformation system, signal transduction, mitochondrial respiration chain, electron transfer chain, and oxidation–reduction system.^16^ Copper imbalance can lead to severe physical disorders, such as Wilson's disease and Menke's disease.^19^, ^20^ Growing evidence has shown that copper levels are related to several types of malignant tumors, with a positive correlation to tumor size, stage, and poor prognosis. This may be due to copper's important role in promoting tumor cell proliferation, angiogenesis, and metastasis.^21^ Similarly, in OC patients, copper levels have been found to obviously increase in the primary tumor, blood serum, and malignant ascites.^22^, ^23^ Copper in ascitic fluid could enhance VEGF expression and promote angiogenesis,^24^ suggesting that copper played an important role in OC progression. However, the correlation between OC and copper metabolism‐related cuproptosis remains unclear. Herein, we explored the relationship between OC and cuproptosis and demonstrated that Elesclomol‐Cu treatment could induce cuproptosis in OC cells, inhibiting their proliferation, migration, and invasion. Interestingly, we also found that even a brief 2‐h exposure to Elesclomol‐Cu resulted in long‐term impairment of cell malignancy, indicating the sustained impact of cuproptosis on OC cells. This study unveiled the novel role of cuproptosis in inhibiting tumor cells, which may provide an effective therapeutic approach for OC.

Considering the crucial role of key regulator FDX1 in cuproptosis, we further explored its potential mechanism in OC cells. Our results showed that OC cells with lower FDX1 expression exhibited higher malignancy and greater resistance to Elesclomol‐Cu treatment, leading to poorer therapeutic effects on cuproptosis. In contrast, high expression of FDX1 inhibited the proliferation, migration, and invasion abilities of OC cells and also increased their sensitivity to cuproptosis. Similarly, we found that Elesclomol pulse treatment in vivo significantly inhibited OC tumor growth and increased FDX1 expression within the tumor. This suggests that those OC tumors with higher FDX1 levels may benefit from clinical cuproptosis treatment. However, our results of clinical samples showed that FDX1 expression in OC tumors was generally lower than in normal ovary tissues. These findings suggest that while cuproptosis represents a promising therapeutic target, a relatively higher expression level of FDX1 in OC tissues is crucial for achieving a positive treatment response.

Until now, platinum‐based chemotherapy remains crucial in OC treatment. The sensitivity of OC patients to platinum‐based drugs is a major influencing factor for their prognosis.^25^ Growing evidence has shown that copper transport proteins obviously influence the sensitivity of platinum‐based drugs. For example, the deficiency of the copper influx protein SLC31A1 reduces cellular uptake of cisplatin, carboplatin, and oxaliplatin,^26^ while overexpression of copper export pumps ATP7A and ATP7B leads to platinum resistance in OC both in vivo and in vitro.^27^, ^28^ This is because copper transport proteins are also involved in platinum transmembrane transport.^29^ In our research, we explored the effect of cuproptosis and cisplatin in OC for the first time and found that cuproptosis could enhance the killing efficacy of cisplatin both in vivo and in vitro. Based on previous studies showing that Elesclomol promotes copper accumulation in the cytoplasm,^3^ we hypothesize that Elesclomol may activate copper transport proteins and simultaneously facilitate the influx of both copper and cisplatin, increasing intracellular cisplatin concentrations and enhancing its cytotoxic effect on OC cells.

Elesclomol, also known as STA‐4783, was initially used as a chemotherapeutic adjuvant for metastatic melanoma. Several clinical trials had explored Elesclomol, alone or in combination with paclitaxel, for treating various solid tumors and demonstrated the good safety of those treatments. However, except for a small clinical trial in stage IV melanoma patients, Elesclomol monotherapy or combination therapy failed to produce the expected clinical response.^30^ In this research, we demonstrated in cell experiments that short‐term exposure to Elesclomol‐Cu induces cuproptosis and significantly reduces OC cell viability. Meanwhile, we also proved that repetitive exposure to Elesclomol could inhibit OC tumor growth in vivo, suggesting that repetitive treatment with Elesclomol may have a good therapeutic effect on OC patients. Additionally, we further revealed that Elesclomol could enhance the killing effect of cisplatin in combination treatment. Notably, Elesclomol treatment alone did not cause serious damage to nude mice, whereas the combination therapy helped partially reverse the side effects of weight loss and kidney injury caused by cisplatin. These results suggest that cuproptosis can amplify the cytotoxic effect of cisplatin, and the combination of Elesclomol and cisplatin may be a promising therapy for OC. Further experiments of this combination for OC treatment should be considered.

Through RNA sequencing, we discovered that cuproptosis can activate the cholesterol biosynthesis pathway and increase total cholesterol levels in OC cells. While many previous researches have shown that cholesterol promotes OC malignancy,^31^ our results indicate that the cytotoxic effect of cuproptosis upon OC cells is decreased in the absence of cholesterol. This is an interesting discovery. Currently, the potential relationships between cuproptosis and cholesterol metabolism remain unclear, but many studies have highlighted the complex interactions between copper and cholesterol metabolism. Huster et al. found that elevated copper inhibited cholesterol biosynthesis and decreased cholesterol levels. Kim et al. found that dietary copper deficiency activated the cholesterol biosynthesis pathway and led to hypercholesterolemia in rats. On the other side, FDX1, the key regulator of cuproptosis, also plays an important role in cholesterol biosynthesis. Mohibi et al. have demonstrated that the knockdown of FDX1 in human colon cancer cells significantly increased intracellular cholesterol levels.^32^ These evidences fully strongly suggest the potential interactions between copper metabolism, cholesterol metabolism, and cuproptosis, which require further experimental validation.

In summary, our findings provide solid evidence that copper with Elesclomol can induce cuproptosis through FDX1 regulation and significantly inhibit OC tumor progression in vivo and in vitro. We also demonstrate that cuproptosis can enhance cisplatin toxicity, and the combination of Elesclomol and cisplatin offers better treatment effects. Moreover, we found that cuproptosis influences cholesterol biosynthesis in OC cells, with cholesterol playing a crucial role in its cytotoxic effect. These results highlight the potential of cuproptosis as a therapeutic strategy, offering a novel approach for OC treatment.

## AUTHOR CONTRIBUTIONS

Qiaojian Zou designed the study, conducted experiments, and wrote the manuscript under the guidance of Shuzhong Yao and Junxiu Liu. Yili Chen and Duo Liu completed animal experiments and partial cell experiments. Chunyu Zhang and Qiuwen Mai helped with clinical specimen collection. Xiaojun Wang, Xiaoying Lin, and Qianrun Chen participated in data analysis and language editing. Mengxun Wei and Chudan Chi arranged the figures and tables. Qiqiao Du and Junxiu Liu contributed to the revision of the manuscript. All authors were involved in drafting and revising the manuscript.

## DISCLOSURES

The authors declare no conflicts of interest.

## ETHICS APPROVAL AND CONSENT TO PARTICIPATE

All clinical samples involved were approved by the Ethical Review Committee of the First Affiliated Hospital of Sun Yat‐sen University and obtained according to the Declaration of Helsinki. Each patient signed a written informed consent for all the procedures. All experimental animal procedures were approved by the Institutional Animal Ethics Committee of Zhiyuan, Guangdong, China.

## Supporting information


Figure S1.

Figure S2.

Table S1.

## Data Availability

The data that support the findings of this study are available on request from the corresponding author.
