# Supplementary figures and images for: Cuproptosis inhibits tumor progression and enhances cisplatin toxicity in ovarian cancer
Source: FASEB J. 2025 Mar 22;39(6):e70484. doi: 10.1096/fj.202500047R (PMC11929041; doi:10.1096/fj.202500047R)

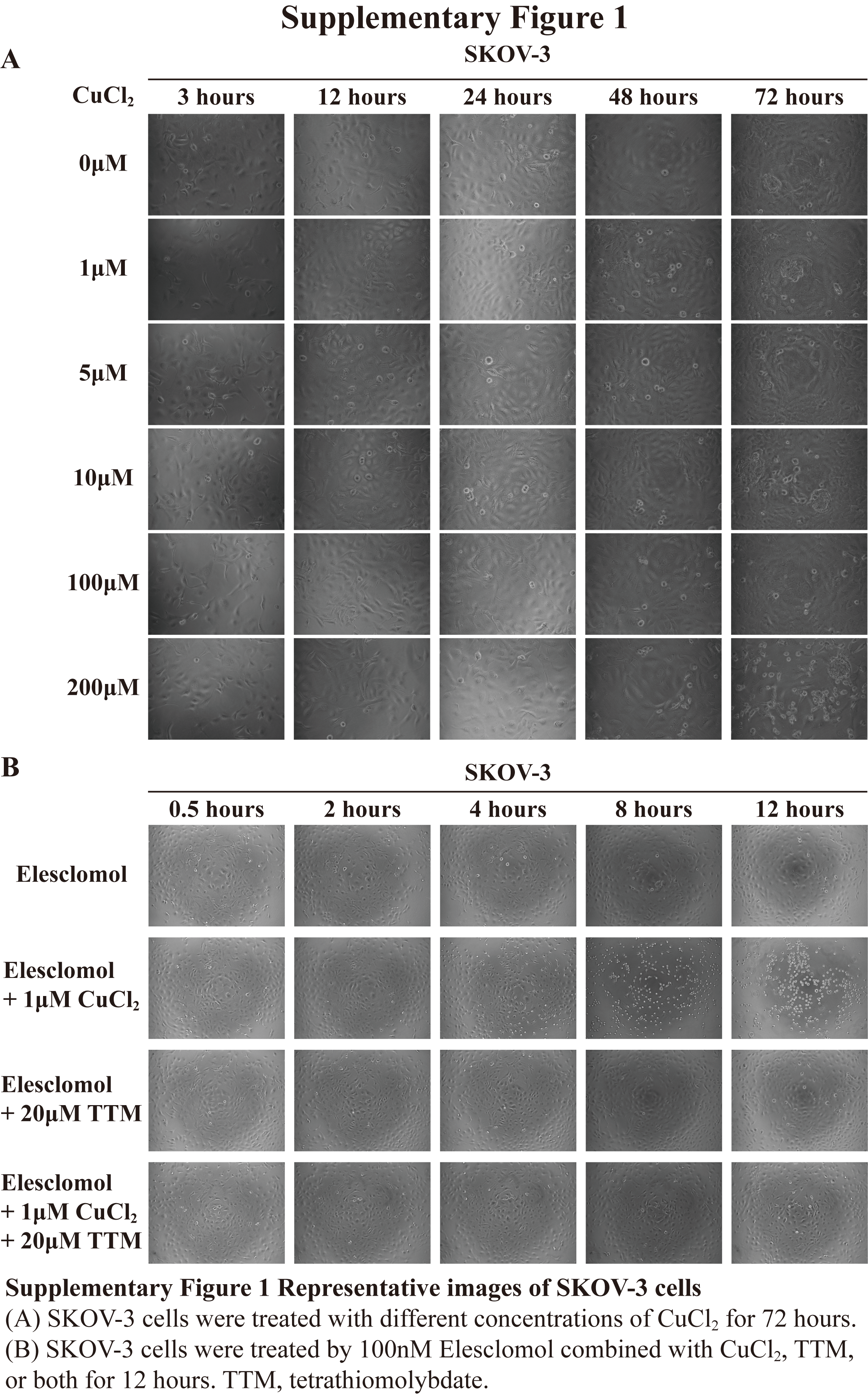

Supplement: Supplementary file 1 — Figure S1. [file FSB2-39-e70484-s002.tif]

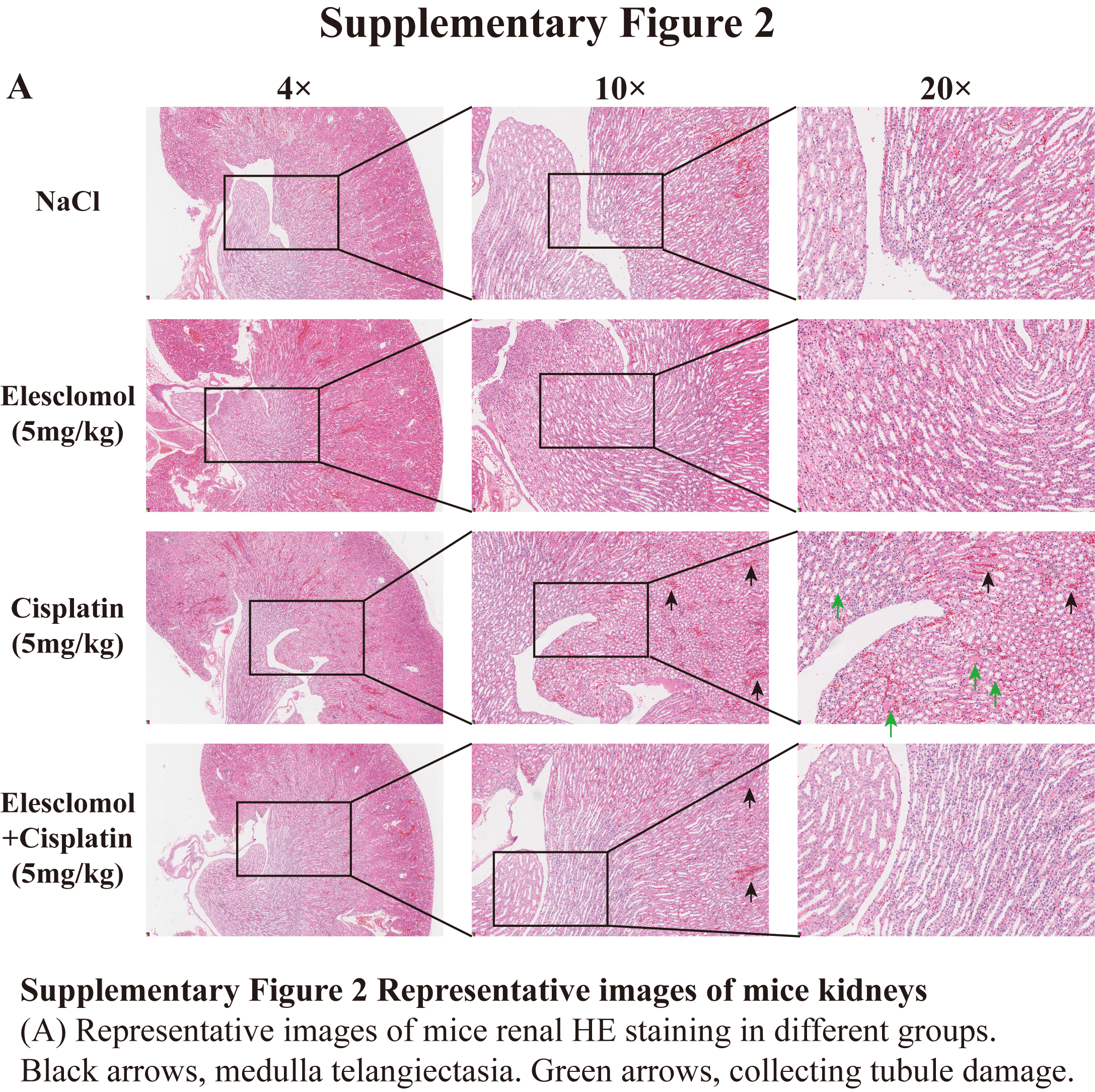

Supplement: Supplementary file 2 — Figure S2. [file FSB2-39-e70484-s001.tif]
