# Supplementary material for: Cuproptosis inhibits tumor progression and enhances cisplatin toxicity in ovarian cancer
Source: FASEB J. 2025 Mar 22;39(6):e70484. doi: 10.1096/fj.202500047R (PMC11929041; doi:10.1096/fj.202500047R)
Supplement: Supplementary file 3 — Table S1. [file FSB2-39-e70484-s003.docx]

**Table S1: Reagents or antibodies in this study**

| **Reagents and antibodies** | **Source** | **Identifier** |
| --- | --- | --- |
| Elesclomol | MedChemExpress | HY-12040 |
| Ammonium tetrathiomolybdate (TTM) | Sigma-Aldrich | 323446 |
| Copper (II) chloride (CuCl_2_) | Macklin | C805300 |
| Zinc chloride (ZnCl_2_) | Macklin | Z820755 |
| Iron (II) chloride (FeCl_2_) | Macklin | I811771 |
| Iron (III) chloride (FeCl_3_) | Macklin | I811935 |
| Magnesium chloride (MgCl_2_) | Macklin | M813913 |
| Cobalt (II) chloride (CoCl_2_) | Macklin | C804815 |
| Nickel (II) chloride (NiCl_2_) | Macklin | N829833 |
| Cisplatin | TargetMol | T1564 |
| Methyl-β-cyclodextrin (M-β-CD) | MedChemExpress | HY-101461 |
| Rabbit anti-FDX1 (1:2000 for WB, 1:200 for IHC) | Proteintech | 12592-1-AP |
| Rabbit anti-Ki67 (1:5000 for IHC) | Proteintech | 27309-1-AP |
| Rabbit anti-LIAS (1:1000) | Proteintech | 11577-1-AP |
| Rabbit anti-HSP70 (1:2000) | PTM-BIO | PTM-5684 |
| Rabbit anti- Caspase 3/P17/P19 (1:1000) | Proteintech | 19677-1-AP |
| Mouse anti-β-tubulin (1:10000) | Proteintech | 66240-1-IG |
| Mouse anti-GAPDH (1:10000) | Proteintech | 60004-1-IG |
| Goat Anti-Mouse IgG (1:10000) | Proteintech | SA00001-1 |
| Goat Anti-Rabbit IgG (1:10000) | Proteintech | SA00001-2 |

**Table S2: Sequence of siRNAs in this study**

| **Gene symbol** | **Sense（5’ to 3’）** | **Antisense（5’ to 3’）** |
| --- | --- | --- |
| NC | UUCUCCGAACGUGUCACGUTT | ACGUGACACGUUCGGAGAATT |
| FDX1-1 | GUGAUUCUCUGCUAGAUGUTT | ACAUCUAGCAGAGAAUCACTT |
| FDX1-2 | CUAACAGACAGAUCACGGUTT | ACCGUGAUCUGUCUGUUAGTT |

**Table S3: Sequence of primers in this study**

| **Gens symbol** | **Forward（5’ to 3’）** | **Reward（5’ to 3’）** |
| --- | --- | --- |
| FDX1 | CCTGGCTTGTTCAACCTGTCA | CCAACCGTGATCTGTCTGTTAGTC |
| HMGCS1 | GATGTGGGAATTGTTGCCCTT | ATTGTCTCTGTTCCAACTTCCAG |
| IDI1 | AACTAGCAGAGCAAGGTTCA | TAGACAGTGGTCCTCAGTTG |
| HSD17B7 | TGGGATCATGCCTAATCCACA | CCAGTTCCCGAATCAGGATAAAA |
| HMGCR | TGAGATCTGGAGGATCCAAGG | AGGATGGCTATGCATCGTGT |
| CYP51A1 | TCTCGTTCCGTCGATTGGGA | GCTATGGCATGCCCAAGGAA |
| EBP | GAGCCCGAACTAGGGATGTG | AGAGAAGAGGCCAGCCAGTA |
| DHCR7 | ACTTTAGCCGGTTGAGAAGGA | AGCTGTACTGGTCACAAGCC |
| SC5D | ATGGAGAGACTTCAGCGCCT | ATCTTCTGGCCATGTGGCTG |
| DHCR24 | CCTGCACACCTTCCAAAACG | CTCTCGCAGCTTGTGGTACA |
| NSDHL | AGGAGTTTTGGAAGTGAGAGAAA | TTCAGAGCTGGGTACAGATCC |
| TM7SF2 | GTCGCCTGCGCTATCCTATTA | TGCGCCTTCATGTAGAGAAAGA |
| GGPS1 | TGGATTAGCAGTAGGTCTCATGC | CCCAAGTGTATTAAGTAGCGGTT |
| SQLE | TGACAATTCTCATCTGAGGTCCA | CAGGGATACCCTTTAGCAGTTTT |
| MVK | GGAGCAAGGTGATGTCACAAC | CGGCAGATGGACAGGTATAAGT |
| INSIG1 | ATCCAGAGGAATGTCACTCTCTT | AGGGGTACAGTAGGCCAACAA |
| FDFT1 | CCACCCCGAAGAGTTCTACAA | TGCGACTGGTCTGATTGAGATA |
| PPARG | ACCAAAGTGCAATCAAAGTGGA | ATGAGGGAGTTGGAAGGCTCT |
| PPARA | CCTGTCTGCTCTGTGGACTC | TGAAAGCGTGTCCGTGATGA |
| ABCA1 | ACCCACCCTATGAACAACATGA | GAGTCGGGTAACGGAAACAGG |
| ABCG5 | TGGACCAGGCAGATCCTCAAA | CCGTTCACATACACCTCCCC |
| NR1H2 | AGAACTAATGATCCAGCAGTTGG | TTGCTTAGCGAAGTCCACGAT |
| LCAT | ACCTGGTCAACAATGGCTACG | TAGAGCAAGTGTAGACAGCCG |
| ABCB1 | ACTCACTTCAGGAAGCAACC | CGAATGAGCTCAGGCTTCCT |
| ACAT2 | ACCCGCCGGTTCCTTTTG | ACCCACACTGGCTTGTCTAA |
| ACAT1 | TACCAGAAGTAAAGCAGCATGG | TCATTCAGTGTACTGGCATTGG |
| SOAT1 | GAAGTTGGCAGTCACTTTGATGA | GAGCGCACCCACCATTATCTA |
| NPC1L1 | CTGGTATCACTGGAAGCGAGT | CACGCGGGTCACATTGATGA |
| MYLIP | AAACCTGAGAAACCGGATCTCC | GCTCCACGAAGAACTTGACTCTA |
| LDLRAP1 | GGGCGGAAAGTTTTTCCTGA | GCGTGTCTGTCCAGTTCTCA |
| LDLR | CACAACCAGGACGGCTACAG | AGATGTTCACGCCACGTCAT |
| PCSK9 | ATGGTCACCGACTTCGAGAAT | GTGCCATGACTGTCACACTTG |
| β-Actin | CATGTACGTTGCTATCCAGGC | CTCCTTAATGTCACGCACGAT |
